# Dynamic Splitting Tensile Behaviour of Concrete Confined by Natural Flax and Glass FRP

**DOI:** 10.3390/polym14204424

**Published:** 2022-10-19

**Authors:** Wenjie Wang, Zonglai Mo, Yunpeng Zhang, Nawawi Chouw

**Affiliations:** 1 Jiangsu Key Laboratory of Engineering Mechanics, School of Civil Engineering, Southeast University, Nanjing 211189, China; 2 School of Mechanical Engineering, Nanjing University of Science and Technology, Nanjing 210094, China; 3 Department of Civil and Environmental Engineering, The University of Auckland, Auckland 1142, New Zealand

**Keywords:** flax-fibre-reinforced polymer, glass-fibre-reinforced polymer, dynamic splitting tensile behaviour, strain rate effect, dynamic increase factor, confinement effectiveness

## Abstract

Flax fibre has been used to reinforce concrete composite, but its dynamic properties have not been thoroughly studied. This study investigates the dynamic splitting tensile properties of plain concrete (PC) confined by flax-fibre-reinforced polymer (FFRP) and glass-fibre-reinforced polymer (GFRP). The dynamic splitting tensile tests were carried out on PC, FFRP-PC, and GFRP-PC cylinder specimens by the high-speed servo-hydraulic machine, with the impact-induced strain rates ranging from 0.1 to 58 s^−1^. The effect of the FRP confinement, FRP thickness and strain rate on the dynamic splitting tensile behaviour were assessed. The results indicated that similar confinement effectiveness of FFRP and GFRP is observed. The dynamic tensile strength of 1- and 2-layer FFRP-PC increased by 29% and 67%, and the one- and two-layer GFRP-PC increased by 32% and 84%, respectively. FFRP-PC and GFRP-PC cylinders showed less sensitivity to the strain rate compared with PC. The empirical relationship between the tensile DIF and strain rate for PC, FFRP-PC and GFRP-PC was proposed based on experimental data. The proposed model was developed to predict the dynamic splitting tensile strength. The results suggested the potential of FFRP composites applied into concrete structures under extreme dynamic loadings.

## 1. Introduction

Fibre-reinforced polymer (FRP) composites as external confinement jackets are growing in popularity to reinforce concrete structures in civil infrastructure, construction and marine applications [1,2,3,4]. It is widely accepted that FRP composites are excellent in the characteristics of high specific tensile and compressive strength, controllable electrical conductivity, low thermal expansion coefficient, good strain ductility, energy dissipation capacity and the production suitability of complex shape materials [5,6,7]. Hence, FRP composites become wider choices for engineering applications as a replacement for conventional materials, which are specially applied for retrofitting, rehabilitating or strengthening materials in extreme conditions such as low or high temperature [8,9,10], seismic area [11,12,13,14] and impact-resistant infrastructure [15,16]. 

Carbon-fibre-reinforced polymer has been widely used in both steel and concrete column structures [17,18]. Wang et al. [19] presented the behaviour of concrete-filled thin-walled steel tubular (CFST) columns partially wrapped by carbon-fibre-reinforced polymer (CFRP) under eccentric compression. The results indicated that the influencing factors for compressive strength mainly include load eccentricity, CFRP confinement factor, steel strength, core concrete strength and CFRP strength. Cao et al. [20] experimentally studied the axial compressive behaviour of CFRP confined expansive concrete columns and developed a calculation model for stress by considering the effect of prestress. Glass-fibre-reinforced polymer-confined concrete [21,22] and aramid-fibre-reinforced polymer-confined concrete [23,24] have also been studied, and it was found that they are effective in enhancing the loading capacity and deformation of the concrete. The above studies indicate that these synthetic-fibre-reinforced polymer-confined types of concrete can effectively improve the static properties by enhancing the axial compressive strength, strain and loading capacity of the concrete columns.

With the increasing attention paid to natural fibres, there is a growing number of studies on natural-fibre-reinforced polymer-strengthened concrete [25,26,27,28,29,30,31]. Yan et al. [25] experimentally investigated the compressive strength of flax-fibre-reinforced polymer- (FFRP) tube-encased coir-fibre-reinforced concrete (CFRC). The effect of FFRP tube thickness and coir fibre inclusion was discussed by analysing the axial stress–strain response, confinement performance, lateral load–displacement response, bond behaviour and failure modes. The study on FFRP and steel spiral dual-confined recycled aggregate concrete (SR-RAC) was performed by Huang et al. [26] and it was concluded that the FFRP-SR-RAC specimens possessed higher compressive strength and failed in a ductile manner compared with the unconfined specimens. In the study by Jirawattanasomkul et al. [28], three natural fibres—jute, hemp and cotton FRP-confined cylinders—were discussed. The results indicated that natural FRP is effective and suitable to enhance the confinement effect of concrete, with the jute fibre having the best performance. In addition, empirical equations were investigated to predict the peak compressive strength of the natural-FRP-confined concrete. Another study on jute-FRP-confined concrete by Gao et al. [29] also revealed that the prefabricated JFRP tube could generate a significant enhancement in the compressive strength and deformation ability of recycled aggregate concrete and sisal-fibre-reinforced recycled-aggregate concrete. It can be concluded that flax and jute have a high profile among all natural fibres, which have been increasingly applied in concrete structures, and performed well, showing great potential to be applied in civil structural construction.

In terms of the dynamic properties, many studies focused on the compressive behaviour of FRP-confined concrete, mainly focusing on carbon [32,33,34,35], glass [36,37,38,39] and aramid fibres [40,41,42,43]. Uddin et al. [32] compared the energy absorption of two different confinement materials, prefabricated polypropylene jacket and CFRP, to strengthen concrete cylinders. The results showed that prefabricated polypropylene jacket concrete specimens performed better. Huang et al. [35,37,44] carried out a series of experimental tests to explore the dynamic behaviour of normal concrete or ultra-high-performance concrete confined by different FRP jackets, such as glass and carbon fabrics. Song et al. [42] studied the behaviour of aramid-FRP-wrapped concrete under repeated impacts. Under multiple impacts with a mean strain rate of 50 s^−1^, the specimens maintained their integrity, showing good impact resistance. Moustafa et al. [45] investigated the effect of strain rate on the properties of rubberized concrete confined by GFRP. Their results showed that the compressive strength increased with strain rate and the layer number of GFRP confinement. It is observed that almost all FRP-confined concrete showed an obvious strain rate effect under different dynamic compressive loadings. However, the behaviour of FRP-confined concrete under dynamic compressive and tensile loading can be very different [46]. 

Some studies reported on the dynamic splitting tensile behaviour of fibre-reinforced concrete [47,48,49,50]. For example, Xie et al. [47] applied a split Hopkinson bar (SHPB) loading system and digital image correlation (DIC) technology to investigate the dynamic splitting tensile properties of basalt-fibre-reinforced concrete (BFRC). The effects of basalt fibre content, loading level on the dynamic tensile properties, as well as energy dissipation and damage pattern were discussed. It was concluded that the dynamic tensile strength and dynamic increase factor (DIF) had obvious connections to strain rate. Hassan and Wille [49] investigated the dynamic tensile behaviour of steel-fibre-reinforced ultra-high-performance concrete (SF-UHPC) by a modified split-Hopkinson tensile bar system with an average strain rate from 10 and 156 s^−1^. It was observed that increasing fibre volume reduced the strain rate sensitivity. The DIF equations for the tensile strength of UHPC reinforced with a 1–4% volume fraction of straight, short and smooth steel fibres were also proposed.

However, very few studies have mentioned the dynamic splitting tensile properties of FRP-confined concrete. Huang et al. [46] investigated the impact resistance of CFRP-confined UHPC under laterally applied splitting loading. The UHPC cylinders were wrapped with CFRP jackets with different thicknesses, and the split Hopkinson pressure bar apparatus was applied to conduct the tests with the strain rate ranging from 2 to 17 s^−1^. The results indicated that the UHPC exhibited strong strain-rate dependency under the dynamic tensile loading, whereas the CFRP confinement can reduce the strain-rate sensitivity. A proposed model to predict the dynamic splitting tensile strength of CFRP-confined UHPC was developed. It was also mentioned that the performance of FRP- confined concrete was different when it was subjected to compressive and tensile loading.

The flax fibre, a potential natural material that could be applied in impact-resistant constructions, has been combined with concrete in recent years to strengthen the concrete structures [51]. The compressive and flexural behaviour of flax-FRP-confined concrete has been studied [52], whereas the dynamic splitting tensile behaviour has not been reported. It is important for a material to be comprehensively studied before it can be applied, especially for structures which could be subjected to extreme loadings. Hence, in this study, the authors focus on the dynamic splitting tensile properties of FFRP-PC and GFRP-PC at different strain rates, with results that are compared with the previous study on the dynamic compressive properties of FFRP-PC and GFRP-PC [53]. The dynamic splitting tensile tests were carried out by the high-speed servo-hydraulic machine, with the impact-induced strain rates ranging from 0.1 to 60 s^−1^. The effect of the FRP confinement, FRP thickness and strain rate on dynamic splitting tensile strength was studied by discussing the tensile stress, dynamic increase factor (DIF) and damage pattern. 

## 2. Materials and Methods

### 2.1. Materials 

The plain concrete (PC), flax-fibre-reinforced polymer-confined PC (FFRP-PC) and glass-fibre-reinforced polymer-confined PC (GFRP-PC) were prepared. PC was composited of cement, water, sand and gravel with a mix ratio by mass of 1:0.46:2.1:2.23, respectively. The sand and gravel were sourced from the Winstone Aggregates Helensville Plant, New Zealand. 

**Figure 1:**
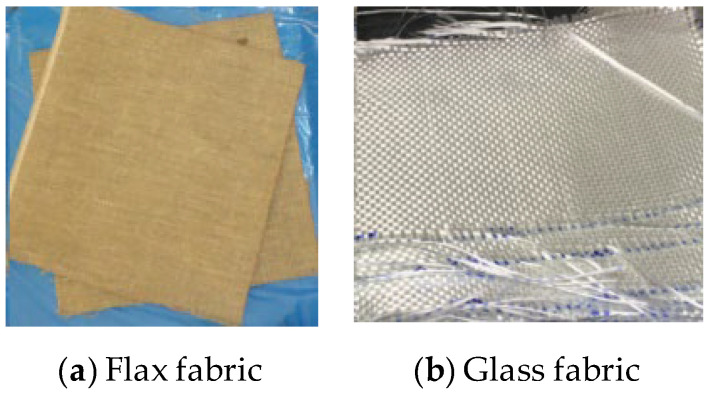
Flax and glass fabric.

The flax fibre BL-550 is a woven balanced flax fabric (Figure 1a), with a fibre yarn density of 1.43 g/cm^3^ and a tensile strength of about 145 MPa. The E-glass fibre WR-400 is a woven and twill glass fabric with a density of about 2.54 g/cm^3^. The epoxy resin used is the SP High Modulus Prime 20LV and its slow hardener, with a mix ratio by weight of 100:26, respectively. The mechanical properties of the epoxy system, FFRP and GFRP laminates are listed in Table 1. More mechanical properties of FFRP and GFRP composites can be found in previous studies [54,55].

### 2.2. Specimens Preparation 

**Figure 2:**
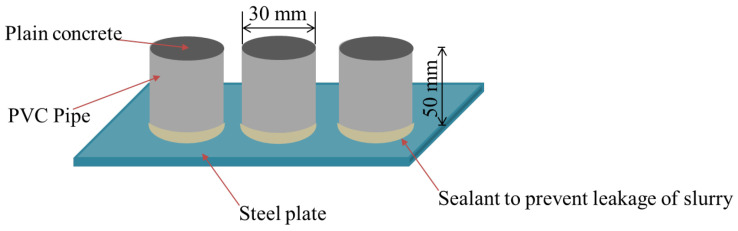
Casting procedure of PC cylinder specimens.

For PC cylinder specimens, a dimension of 50 mm × 30 mm was prepared both for quasi-static and dynamic tests. It is noted that the cylinder specimen dimensions were referred to split-Hopkinson pressure bar (SHPB) tests by some previous studies [56,57,58]. The casting procedure is summarised as follows: (1) The polyvinylchloride (PVC) pipe with an inner diameter of 30 mm was prepared as mould, which was cut into short tubes with a height of 50 mm for casting the concrete specimen; (2) the bottom ends of the PVC short tube moulds were placed on a big piece of flat steel plate, and the contact area between the PVC pipe and the steel plate was sealed with sealant to prevent leakage of slurry; (3) the concrete mixture was prepared using a blender and poured into the moulds and compacted evenly and (4) they were demoulded after 24 h and cured in a concrete curing room at a stable temperature of 23.0 ± 2.0 °C and a relative humidity of 95% for 28 days. The detailed casting procedure is displayed in the sketch in Figure 2.

**Figure 3:**
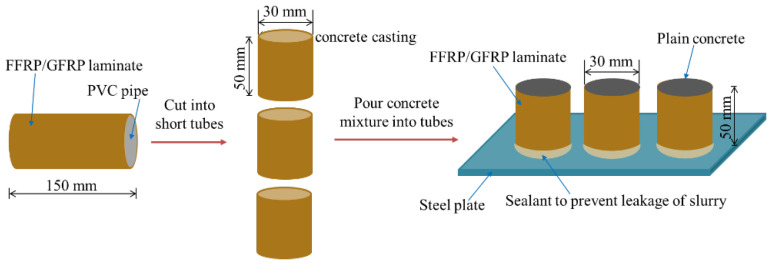
Casting procedure of FFRP-PC and GFRP-PC cylinder specimens.

**Figure 4:**
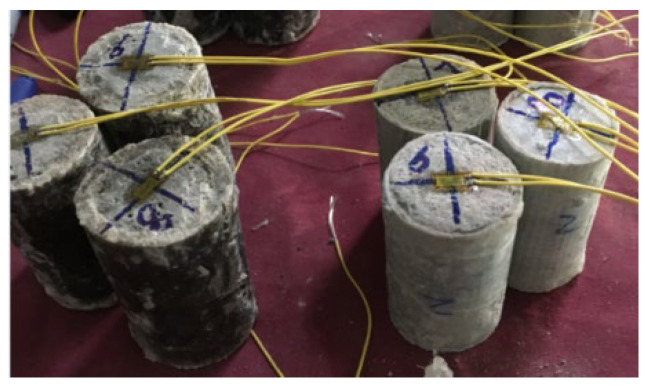
FFRP-PC and GFRP-PC cylinder specimens.

For the FFRP- and GFRP-confined PC cylinders, flax and glass fabric sheets were cut into appropriate lengths. A length of 200 mm was finally decided after trying different tube lengths ranging from 50 to 300 mm, where the range of 100–200 mm was found to be better for the concrete quality. The one- and two-layer FFRP and GFRP laminates were respectively prepared by the hand lay-up method. The fabric was firstly saturated with epoxy on the surfaces using brushes or rollers and wrapped tightly around the PVC tubes, whose surfaces were coated with a lubricating oil film to facilitate the demoulding. The FFRP and GFRP tubes were then pulled out from PVC tubes after 4 h of curing and then dried for another 72 h at normal temperature. Plain concrete was cast and poured into the FFRP and GFRP tubes. The detailed casting procedure of FFRP/GFRP-PC is displayed in the sketch in Figure 3. The FFRR-PC and GFRP-PC cylindrical specimens are shown in Figure 4. The test matrix of the test specimens is listed in Table 2. It is noted that all specimens were prepared at the Civil Material Test Lab at the University of Auckland under the same environmental conditions. 

### 2.3. Testing Apparatus

#### 2.3.1. Quasi-Static Test

The quasi-static compressive and tensile tests were carried out according to ASTM Standard C39/C39M-10 [59] and C496/C496M-11 [60], respectively. The quasi-static split tensile strength can be calculated:

(1) $$f_{t,s}\overset{}{}\;\mathrm{and}\;\underset{}{}f_{tu,s}=\frac{2P}{\pi ld}$$

where *f_t,s_* is the split tensile strength of unconfined PC specimens, *f_tu,s_* is the split tensile strength of the confined specimens and subscript *s* implies a static test. *P* is the maximum load, *d* is the diameter of the cylinder and *l* is the height of the specimen.

#### 2.3.2. Dynamic Splitting Tensile Test

**Figure 5:**
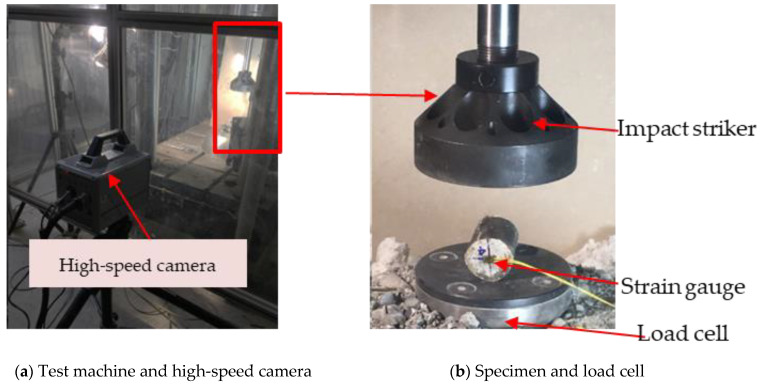
Dynamic split tensile test instrument and setup.

The dynamic splitting tensile test was carried out using a servo-hydraulic Instron VHS 160-20 machine, which has a maximum velocity of up to 25 m/s and a maximum dynamic load of 100 kN, as shown in Figure 5a. In the study, various impact velocities of 0.1, 1, 2.5, 5 and 6.5 m/s were applied at a room temperature of about 20 °C.

The experiment setup for the dynamic splitting tensile test is displayed in Figure 5b. The dynamic force was measured from the piezo load cell, which was installed below the bottom-grip head. Strain gauges were attached to the centre of the specimen to measure the strain development of the core concrete and calculate the strain rate. The data acquisition system with a sampling frequency of 65 kHz was used and a high-speed camera with a frequency of 30,000 fps was utilised to record the damage process. 

## 3. Results

### 3.1. Quasi-Static Mechanical Properties

**Figure 6:**
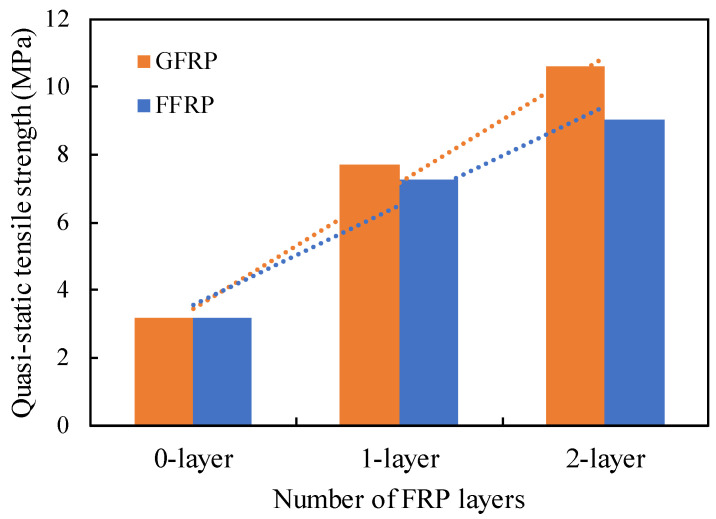
Effect of FRP layers on the quasi-static splitting tensile strength.

Table 3 displays the experimental test results of the PC, FFRP-PC and GFRP-PC under quasi-static compressive and tensile loadings. It is noted that the value in the table is the average result obtained from three samples for each type of specimen and the results of compressive strength are obtained from the author’s previous study [53]. The compressive strength of PC is 33.02 MPa, which value is increased with the confinement of FFRP or GFRP. The compressive strength of one-layer FFRP-PC is 45.29 MPa, whereas the two-layer FFRP confinement has a larger value of 51.11 MPa. Similarly, the compressive strength of GFRP-L1-PC and GFRP-L2-PC is 42.3 and 52 MPa, respectively. For tensile strength, a similar increase tendency is observed. The tensile strength of PC is 3.18 MPa, with the FFRP-L1-PC of about 7.28 MPa and FFRP-L2-PC of about 9.05 MPa. The GFRP-confined PC has an average value of 7.72 MPa and 10.62 MPa for one- and two-layer, respectively. Figure 6 displays the effect of FRP layers on the quasi-static splitting tensile strength. An approximately linear relationship is observed between the numbers of FFRP/GFRP layer and the tensile strength of the corresponding confined PC. The confinement increasing ratio (CIF) [46], the ratio of unconfined PC strength to its corresponding FRP confined PC strength, also indicates the effectiveness of FRP confinement on both compressive strength and tensile strength, with the CIF for compression (CIF_c_) ranging from 1.372–1.548 for one- and two-layer FFRP, respectively, and 1.281–1.842 for one- and two-layer GFRP, respectively. 

**Figure 7:**
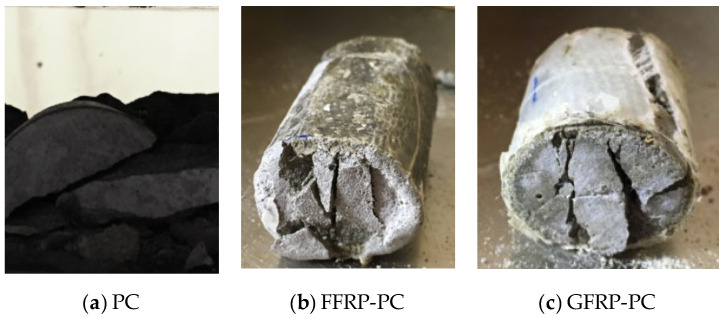
Failure mode under quasi-static split tensile test.

Figure 7 displays the failure modes of PC, FFRP-PC and GFRP-PC specimens under quasi-static split loading. The typical brittle failure of PC is shown in Figure 7a, with the entire specimen crushed into fragments. Compared with PC, FFRP-PC and GFRP-PC specimens keep together, with the crushed concrete core confined by cracked FRP layers, as shown in Figure 7b,c. The damage pattern of FFRP-PC and GFRP-PC is similar. The longitudinal rupture of FFRP and GFRP jackets following concrete core crush was the main failure process of confined concrete under quasi-static splitting loading, which is similar to that under compressive loading [53]. However, the two failure mechanisms of FRP rupture are different between tensile and compressive cases. For the compressive case, the concrete core expands evenly in the hoop direction, whereas the deformation of the concrete core is not uniform, which can be proved by the central cracking. Due to the splitting of the concrete core, the FRP jackets were propped at a position perpendicular to the crack, resulting in the maximum tensile strain of FRP jackets due to stress concentration [46].

### 3.2. Dynamic Splitting Tensile Test Results

#### 3.2.1. Time Histories of Stress 

**Figure 8:**
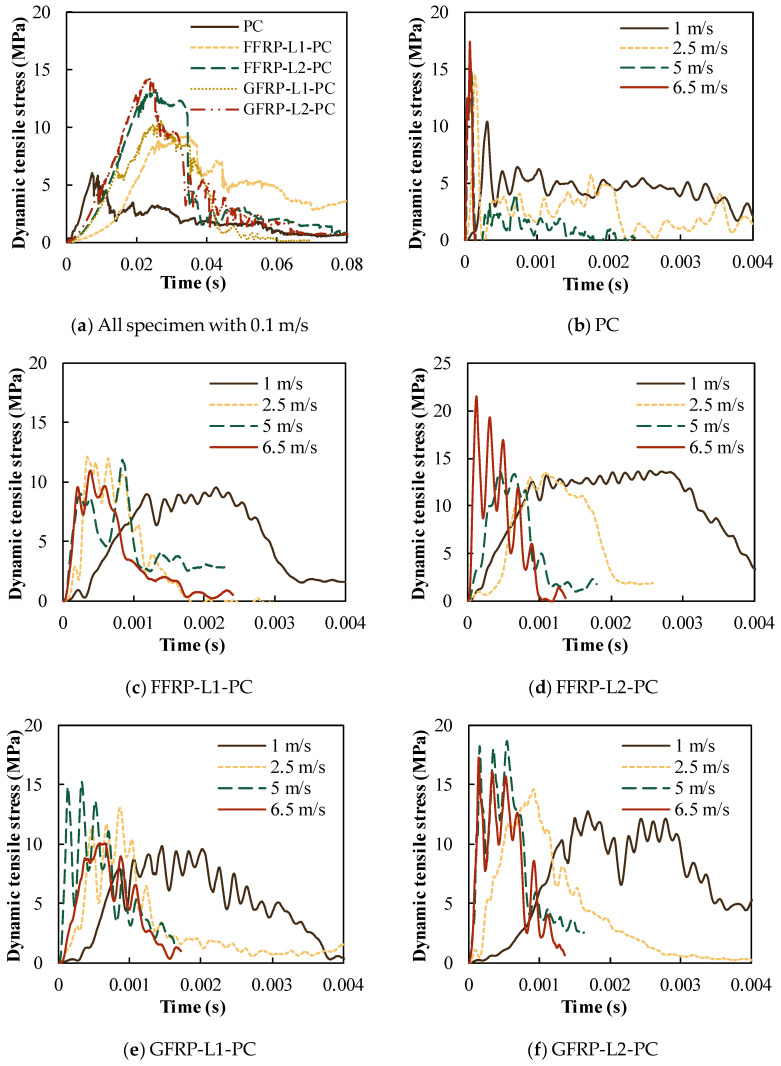
Stress histories of PC, FFRP-PC and GFRP-PC under different speeds.

The stress due to the dynamic tensile load was calculated from Equation (1), with the force recorded from the dynamic load cell. Figure 8 presents the time history of the stress of PC, FFRP-PC and GFRP-PC cylinders under different actuator velocities, respectively. Figure 8a displays the stress–time curves of all types of specimens under the velocity of 0.1 m/s, which corresponds to the strain rate of 0.19–0.38 s^−1^. The strain rate of the specimen was calculated by the secant slope of the strain time history. The stress duration of all specimens with 0.1 m/s of velocity is about 0.08 s. PC specimens reach the peak stress at about 0.008 s with a strain rate of 0.38 s^−1^. By contrast, FFRP- and GFRP-confined PC specimens require a longer time of around 0.03 s to reach the peak stress, with a smaller strain rate of about 0.2 s^−1^. The number of FRP layers shows an obvious influence on the tensile stress. With FFRP confinement, the tensile stress of one- and two-layer confined PC increased by 29% and 67%, respectively. Similarly, the one- and two-layer GFRP- confined PC specimens showed a corresponding increment of 32% and 84% of tensile stress. 

Figure 8b shows the time histories of the stress of PC with the velocities of 1, 2.5, 5 and 6.5 m/s, within the duration of around 0.004, 0.003, 0.002 and 0.0001 s, respectively. The duration became significantly shorter with increasing strain rate, whereas the stress increased with the strain rate. A similar tendency of stress duration can be observed in the FFRP-PC specimens, as shown in Figure 8c,d, and GFRP-PC, shown in Figure 8e,f. This indicates that the stress duration of PC is strain-rate sensitive. A great difference in stress duration was found between PC and FRP-confined PC under the velocity of 6.5 m/s. The duration was about 0.0001 s for PC and 0.0015 s for both FFRP- and GFRP-confined PC, which could be explained by the strain rate difference. The strain rate is around 57.6 s^−1^ for PC and 20 s^−1^ for FRP-confined PC, indicating that the strain rate for PC is much larger than that of confined PC, resulting in the acceleration of the stress time history. This also implies that PC is more sensitive to the strain rate compared with FRP-confined PC under the case of dynamic tensile loading.

#### 3.2.2. Strain Rate Effect on Tensile Behaviour 

The strain rate of the specimen was calculated by the secant slope of the strain time history. The dynamic increase factor (DIF) of splitting tensile strength was calculated by the dynamic tensile strength normalized by the average static tensile strength of the same type of specimen, listed respectively in Table 4, Table 5 and Table 6 for PC, FFRP-PC and GFRP-PC specimens. 

From Table 4, the actuator velocities applied on PC specimens were ranging from 0.1 to 6.5 m/s, with the corresponding average strain rates from 0.38 to 57.6 s^−1^. The dynamic tensile strength increased with the strain rate. The stress at the strain rate of 0.38 s^−1^ was about 7.10 MPa, which is 10.85 MPa at the strain rate of 5.25 s^−1^ and 20.21 MPa at the strain rate of 48.75 s^−1^. The strength has no obvious increment tendency after this strain rate with the velocity of 5 m/s. It indicates that the PC specimens are more sensitive to strain rate when their value is lower than a specific point, i.e., 48.75 s^−1^. For FFRP-confined PC specimens, the tensile stress for the one-layer case increased from 9.13 MPa to 10.93 MPa, with the strain rate changing from 0.21 to 17.05 s^−1^, whereas the stress for the two-layer case increased from 11.07 to 15.33 Mpa, with a strain rate ranging between 0.19 and 20.3 s^−1^. GFRP-confined PC specimens exhibit similar characteristics to FFRP-PC, with a similar strain rate range and tensile strength, as shown in Table 6.

From the test results, the dynamic tensile stress of FFRP-PC and GFRP-PC did not increase with the number of FRP layers. To discuss the effect of the FFRP/GFRP layer on the dynamic tensile strength, the confinement effectiveness (*CE*) [53] is defined as the tensile strength of the confined concrete divided by the unconfined concrete tensile strength:

(2) $$CE=\frac{f_{tc}}{f_{to}}$$

where $f_{tc}$ is the dynamic tensile strength of FFRP/GFRP-PC at different strain rates and $f_{to}$ is the dynamic tensile strength of PC at different strain rates.

**Figure 9:**
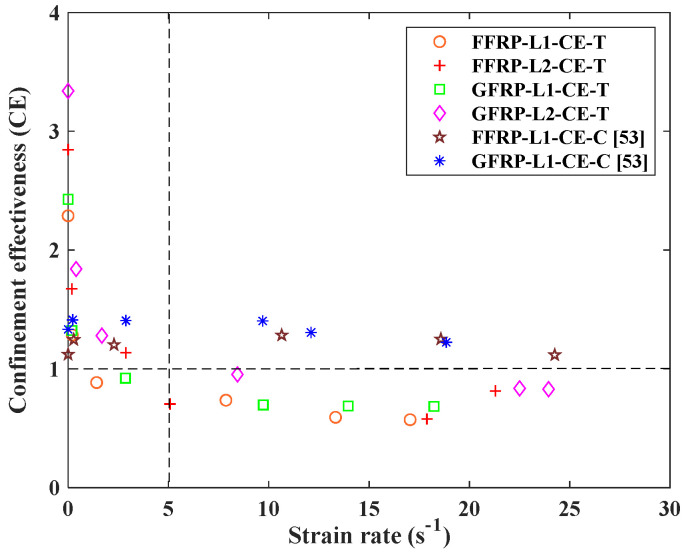
Confinement effectiveness of FFRP/GFRP vs. strain rate.

Figure 9 shows the tendency of *CE* of FFRP/GFRP with strain rate both under tensile and compressive loadings. It can be found that the confinement effectiveness under compressive load is higher than that under tensile load for both FFRP- and GFRP-confined PC. This effectiveness is especially verified at the high strain rate range of larger than 5 s^−1^, with the value of *CE* around 1.3 for the compressive case but below 1.0 for the tensile case. This indicates that both FFRP and GFRP composites could be more effective in enhancing both compressive and split tensile strength at a low strain rate of lower than 5 s^−1^, which can be used as a potential construction material in the low strain rate impact area [53]. At the strain rate of larger than 5 s^−1^, the value of *CE* is larger than 1 for compressive strength, whereas it is smaller than 1 for the tensile strength whatever the FRP layer. This indicates that FRP confinement effectiveness seems weakened for the tensile strength at high strain rates. This could be due to the energy absorption limitation achieved by the FRP-PC material under the splitting tensile load condition, resulting in similar damage at higher different strain rates and FRP layer numbers. 

**Figure 10:**
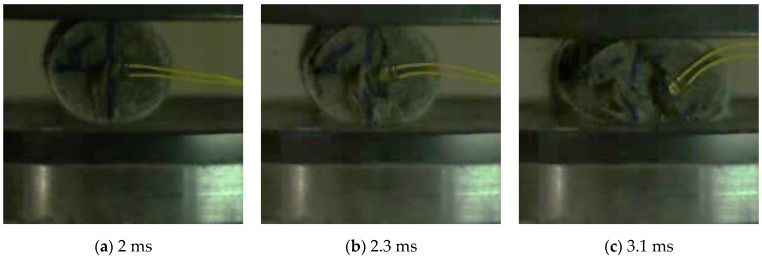
Failure process of GFRP-L1-PC at the strain rate of 8.39 s^−1^.

Considering the damage pattern, it is observed that the damage pattern of FFRP-PC and GFRP-PC is similar, which is not influenced by the strain rate and the FRP layer number. Taking the failure process of GFRP-L1-PC at the strain rate of 8.39 s^−1^ as an example in Figure 10, the damage process under tensile load can be summarised: the crack along the centre of the cross-section concrete core was developed (Figure 10a). The concrete core was split along the crack after reaching the concrete ultimate tensile strain (Figure 10b). The FRP jacket was firstly deformed together with the concrete under pressure (Figure 10b). Then the concrete deformation exceeded the ultimate strain and extruded the FRP jacket, leading to the overall failure of the specimen (Figure 10c). The damage process of the specimen under tensile load is different from that of under compressive load [53]. For the compressive case, with the increase of compression, the deformation gradually increased until failure, with the outer FRP package squeezed, failing the specimen. This difference in the damage process could be a reason for the confinement effectiveness difference between dynamic compression and tension. From the above discussion, it is concluded that the FRP confinement may not be able to enhance the tensile strength significantly under dynamic impact loadings, whereas the FRP jacket can be beneficial to keep the damaged structure’s integrity to reduce the casualties caused by collapses of structures. 

#### 3.2.3. Dynamic Splitting Tensile Strength Models 

**Figure 11:**
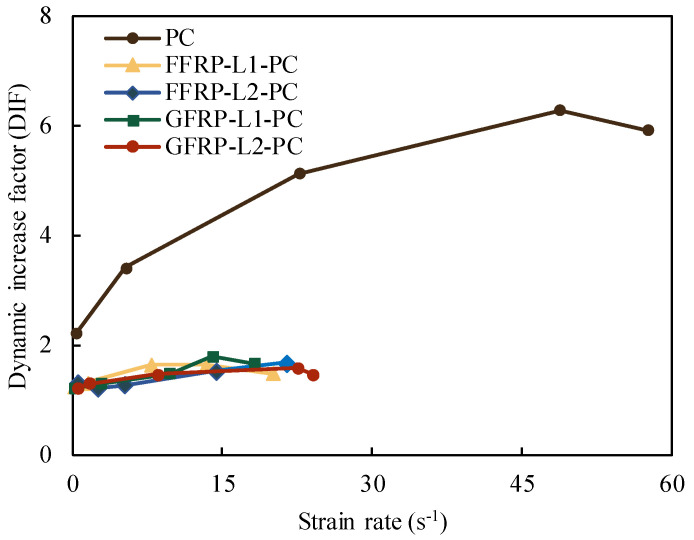
Strain rate effect on the tensile strength of PC, FFRP-PC and GFRP-PC.

The effect of strain rate on the tensile strengths of all types of specimens is illustrated in Figure 11. For different construction materials, the strain rate of PC was higher than that of FFRP- and GFRP-confined PC. The DIF increased with the strain rate regardless of the confinement ratio, which trend was particularly pronounced for PC. Both FFRP- and GFRP-confined PC specimens showed a slow increase in strain rate. It is observed that when the actuator velocity is larger than 5 m/s, the DIF did not increase with the corresponding strain rate. 

To predict the dynamic split tensile strength, the tensile DIF-versus-strain-rate models by CEB Model Code [61] and Malvar and Ross [62] were applied, which were displayed in Equations (3) and (4), respectively.

(3) $$\left\{\begin{array}{c} DIF={\left(\frac{\dot{\varepsilon }}{{\dot{\varepsilon }}_{s}}\right)}^{1.016\delta }\underset{}{}\;\mathrm{for}\;\underset{}{}\dot{\varepsilon }\leq 30\underset{}{}\;s^{-1}\\ DIF=\beta {\left(\frac{\dot{\varepsilon }}{{\dot{\varepsilon }}_{s}}\right)}^{1/3}\underset{}{}\;\mathrm{for}\;\underset{}{}\dot{\varepsilon }>30\underset{}{}\;s^{-1} \end{array}\right.$$

where

$\dot{\varepsilon }$ is the strain rate
${\dot{\varepsilon }}_{s}$ is the quasi-static strain rate, with a value of 3 × 10^−6^

$\log\beta =7.11\delta -2.33,\overset{}{}\delta =1/(10+6f_{c}/f_{co}),\underset{}{}f_{co}=10\;\mathrm{MPa}$

$f_{c}$ is the quasi-static compressive strength.

(4) $$\left\{\begin{array}{c} DIF={\left(\frac{\dot{\varepsilon }}{{\dot{\varepsilon }}_{s}}\right)}^{\delta }\underset{}{}\;\mathrm{for}\;\underset{}{}\dot{\varepsilon }\leq 1\underset{}{}\;s^{-1}\\ DIF=\beta {\left(\frac{\dot{\varepsilon }}{{\dot{\varepsilon }}_{s}}\right)}^{1/3}\underset{}{}\;\mathrm{for}\;\underset{}{}\dot{\varepsilon }>1\underset{}{}\;s^{-1} \end{array}\right.$$

where

$\dot{\varepsilon }$ is the strain rate;
${\dot{\varepsilon }}_{s}$ is the quasi-static strain rate, with a value of 10^−6^;
$\log\beta =6\delta -2,\overset{}{}\delta =1/(1+8f_{c}/f_{co}),\underset{}{}f_{co}=10\;\mathrm{MPa}$;
$f_{c}$ is the quasi-static compressive strength.

**Figure 12:**
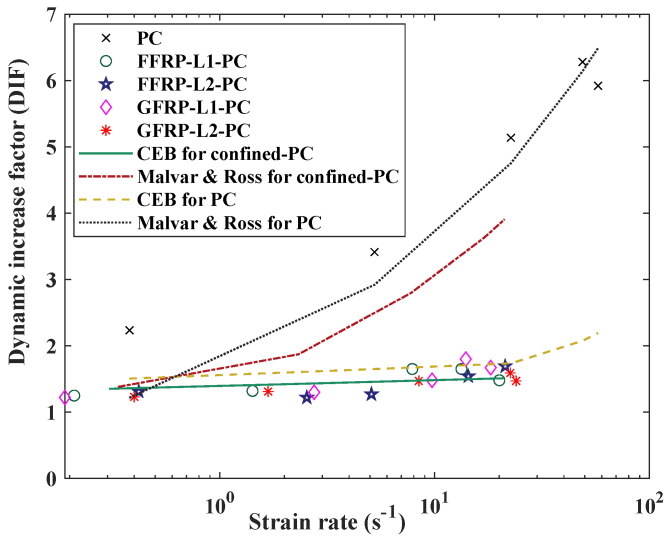
Comparison of the experimental results with current tensile DIF models.

**Figure 13:**
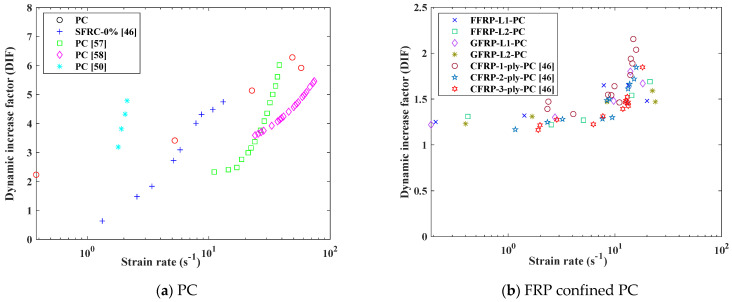
Comparison with existing literature on DIF of PC and FRP-confined PC.

Figure 12 compares the experimental results of the predictions of the two models described in Equations (3) and (4) with the axis strain rate expressed in the logarithm. It is observed that the CEB Model obviously underestimated the DIF of PC, but it could properly predict the variation trend of DIF of FFRP- and GFRP-confined PC with strain rate. Instead, Malvar and Ross’s Model can predict the DIF of PC but overestimated the DIF of FFRP/GFRP-confined PC. For further discussion, the test results from existing literature are reviewed and compared with the current study. Figure 13a compares the test results of PC with the existing literature [50,56,57,58]. It can be found that PC is very strain-rate-sensitive in terms of tensile strength. In the study by Feng et al. [56], the strain rate changed from 1.36 to 13.71 s^−1^, with the DIF increasing from 1.09 to 4.91. Hao et al. [57] concluded that the DIF with a high strain rate can reach a high DIF of more than 6. In the studies by [50,58], the DIF of PC is also around 5 with a high strain rate. These results are consistent with the experimental results in this study. Figure 13b compares the results among FFRP-, GFRP- and CFRP-confined PC. The DIF of CFRP- confined UHPC was studied by Huang et al. [46] through SHPB tests. The results showed that FRP-confined concrete specimens displayed a similar increment trend regardless of the fabric type. Among these three fibre types, one layer of CFRP-confined concrete showed a relatively higher strain rate sensitivity with a higher value of DIF compared with FFRP and GFRP. This could be due to several factors, such as concrete strength, specimen size effect or the material characteristic difference among carbon, glass and flax fibres.

**Figure 14:**
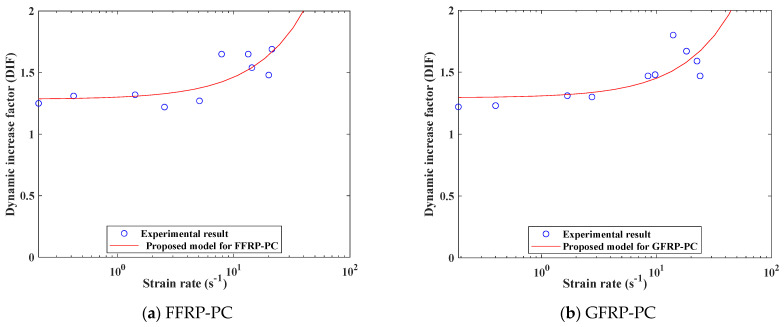
Proposed model for the experimental results.

A new model is developed to predict the tensile DIF for FFRP/GFRP-confined PC. It is widely accepted that a logarithmic relationship exists between strain rate and the DIF for concrete materials [46]. Hence, the best-fit tensile strength DIF-strain rate relationship of FFRP-PC and GFRP-PC are listed in Equations (5) and (6), respectively. As shown in Figure 14, the proposed model performed well in predicting the tensile DIF of the specimens with a strain rate below 30 s^−1^.

(5) $$DIF_{FFRP-PC}=0.01793\log\dot{\varepsilon }+1.283,\underset{}{}\;\mathrm{for}\;\underset{}{}\dot{\varepsilon }\leq 22\underset{}{}\;1/s$$

(6) $$DIF_{GFRP-PC}=0.0158\log\dot{\varepsilon }+1.293,\underset{}{}\;\mathrm{for}\;\underset{}{}\dot{\varepsilon }\leq 25\underset{}{}\;1/s$$

(7) $$f_{tc,d}=(a\frac{\dot{\varepsilon }}{{\dot{\varepsilon }}_{0}}+1)f_{t,s}+bf_{lu}$$

where *f_tc,d_* is the dynamic split tensile strength of FFRP/GFRP-confined PC specimens, and *f_t,s_* is the split tensile strength of the confined specimens. *a* and *b* are undermined constants which characterise the dynamic resistant effect and confinement effect. *f_lu_* is the nominal confinement force, which is determined from the equation:

(8) $$f_{l}=\frac{2E_{frp}t\varepsilon_{frp}}{d}$$

where *E_frp_* and *t* are the elastic modulus and the thickness of the FRP jacket, respectively. *ε_frp_* is the rupture strain of FRP, and *d* is the diameter of the specimen.

Based on the experimental data and the proposed Equation (8), the empirical model is regressed to predict the dynamic tensile split strength, as shown in Equations (9) and (10) for FFRP and GFRP confined PC, respectively.

(9) $$f_{tc,d-FFRP}=(1.11\times 10^{-6}\frac{\dot{\varepsilon }}{{\dot{\varepsilon }}_{0}}+1)f_{t,s}+0.6956f_{lu}$$

(10) $$f_{tc,d-GFRP}=(1.11\times 10^{-6}\frac{\dot{\varepsilon }}{{\dot{\varepsilon }}_{0}}+1)f_{t,s}+0.8064f_{lu}$$

## 4. Conclusions

In this research, the dynamic split tensile properties of PC, flax-fibre-reinforced polymer-confined PC and glass-fibre-reinforced polymer-confined PC composites were experimentally studied. The strain rates ranging from 0.1 to 60 s^−1^ were considered. The results revealed:

(1) Both the quasi-static compressive and splitting tensile strength were significantly improved by the confinement of FFRP and GFRP jackets;

(2) PC, FFRP-PC and GFRP-PC specimens exhibited strain rate dependency. The dynamic increase factor increased with strain rate. FFRP-PC and GFRP-PC showed less sensitivity to the strain rate compared with PC specimens.

(3) FFRP and GFRP showed similar confinement effectiveness for reinforcing concrete strength under dynamic tensile loadings. Also, FFRP-PC and GFRP-PC specimens showed similar damage patterns under both quasi-static and dynamic load conditions.

(4) The empirical models were proposed to predict the DIF of the FFRP-PC and GFRP-PC at different strain rates, respectively; 

(5) The proposed models were developed to predict the dynamic splitting tensile strength of FFRP-PC and GFRP-PC. 

Generally, from the study, it can be found that natural flax fibre shows good potential to be used in civil construction, especially under dynamic conditions. The application of this flax fibre material in civil structures is promising to achieve the advantages of being environmentally friendly and low-cost while satisfying the dynamic-resistant structural properties.

## Figures and Tables

**Table 1:** Mechanical properties of FFRP and GFRP.

Materials	Properties	Value	
**FFRP**	Thickness with one layer (mm)	0.98	
	Static tensile strength (MPa)	29.8	
	Dynamic tensile strength (MPa)	33.29	
	Static tensile strain	0.023	
**GFRP**	Thickness with one layer (mm)	0.62	
	Static tensile strength (MPa)	307	
	Dynamic tensile strength (MPa)	377.1	
	Static tensile strain	0.018	
**Epoxy system**	Elastic modulus (GPa)	3.5	
	Tensile strength (MPa)	73	
	Strain to failure (%)	3.5	
	Cured density (g/cm^3^)	1.144	
	Linear shrinkage (%)	1.765	
	Barcol hardness	27	
	Density (g/cm^3^)	1.123 (20LV Resin)	0.936 (Slow hardener)
	Volume ratio	100 (20LV Resin)	31.4 (Slow hardener)
	Viscosity @20°C (cP)	1010–1070 (20LV Resin)	22–24 (Slow hardener)

**Table 2:** Test matrix of the PC, FFRP-PC and GFRP-PC specimens.

Specimen Type	Actuator Velocity (m/s)	No. of Specimens	Fibre Thickness (mm)
PC	Quasi-static split test	3	-
	0.1	3	-
	1.0	3	-
	2.5	3	-
	5.0	3	-
	6.5	3	-
FFRP-L1-PC ^1^	Quasi-static split test	3	0.96
	0.1	3	0.96
	1.0	3	0.98
	2.5	3	0.92
	5.0	3	1.00
	6.5	3	0.96
FFRP-L2-PC	Quasi-static split test	4	1.89
	0.1	3	1.89
	1.0	3	1.98
	2.5	3	1.78
	5.0	3	1.92
	6.5	3	1.96
GFRP-L1-PC	Quasi-static split test	4	0.67
	0.1	3	0.63
	1.0	3	0.65
	2.5	3	0.72
	5.0	3	0.75
	6.5	3	0.68
GFRP-L2-PC	Quasi-static split test	4	1.33
	0.1	3	1.30
	1.0	3	1.19
	2.5	3	1.22
	5.0	3	1.15
	6.5	3	1.28

^1^ L means the number of FRP layers.

**Table 3:** Experimental results of FFRP/GFRP-confined PC under quasi-static loading.

Specimen Type	Compressive Strength (MPa)	CIF*_c_*	Splitting Tensile Strength (MPa)	CIF*_t_*
PC	33.02 (1.2)	-	3.18 (0.5)	-
FFRP-L1-PC	45.29 (2.3)	1.372	7.28 (0.8)	2.286
FFRP-L2-PC	51.11 (3.0)	1.548	9.05 (1.3)	2.842
GFRP-L1-PC	42.30 (2.8)	1.281	7.72 (0.9)	2.424
GFRP-L2-PC	52.00 (3.9)	1.842	10.62 (1.2)	3.333

[Note] Values in parentheses are standard deviations.

**Table 4:** Experimental results of PC under dynamic tensile loading.

Specimen No.	Velocity (m/s)	Strain Rate (s^−1^)	Maximum Strain	Maximum Load (kN)	Dynamic Splitting Tensile Strength (MPa)	DIF
**PC-01**	0.1	0.38	0.0030	14.26	6.06	1.906
**PC-02**	0.1	0.35	0.0034	17.96	7.62	2.396
**PC-03**	0.1	0.42	0.0031	17.95	7.62	2.396
**Average**	0.1	0.38 (0.036)	0.0032 (0.0002)	16.72 (2.133)	7.10 (0.901)	2.233 (0.142)
**PC-04**	1	4.93	0.0021	24.52	10.41	3.274
**PC-05**	1	5.44	0.0037	26.03	11.05	3.475
**PC-06**	1	5.36	0.0022	26.15	11.10	3.491
**Average**	1	5.25 (0.275)	0.0027 (0.0009)	25.57 (0.908)	10.85 (0.385)	3.413 (0.060)
**PC-07**	2.5	22.38	0.0034	43.34	18.40	5.786
**PC-08**	2.5	22.59	0.0032	34.40	14.60	4.591
**PC-09**	2.5	23.03	0.0024	37.70	16.01	5.035
**Average**	2.5	22.67 (0.332)	0.0030 (0.0005)	38.48 (4.521)	16.34 (1.921)	5.137 (0.302)
**PC-10**	5	40.84	0.0025	46.21	19.62	6.170
**PC-11**	5	57.40	0.0041	50.07	21.26	6.686
**PC-12**	5	48.02	0.0035	46.54	19.76	6.214
**Average**	5	48.75 (8.304)	0.0034 (0.0008)	47.61 (2.140)	20.21 (0.909)	6.356 (0.143)
**PC-13**	6.5	57.45	0.0033	47.03	19.97	6.280
**PC-14**	6.5	70.33	0.0047	41.39	17.57	5.525
**PC-15**	6.5	45.01	0.0033	44.60	18.93	5.953
**Average**	6.5	57.60 (12.66)	0.0038 (0.0008)	44.34 (2.829)	18.82 (1.204)	5.919 (0.189)

[Note] Values in parentheses are standard deviations.

**Table 5:** Experimental results of FFRP-confined PC under dynamic tensile loading.

Specimen No.	Velocity (m/s)	Strain Rate (s^−1^)	Maximum Strain	Maximum Load (kN)	Dynamic Splitting Tensile Strength (MPa)	DIF
**F-L1-01**	0.1	0.25	0.0032	24.95	10.09	1.39
**F-L1-02**	0.1	0.25	0.0040	21.21	8.57	1.18
**F-L1-03**	0.1	0.14	0.0030	21.60	8.73	1.20
**Average**	0.1	0.21 (0.064)	0.0034 (0.0005)	22.59 (2.056)	9.13 (0.835)	1.25 (0.115)
**F-L1-04**	1	1.50	0.0024	22.02	10.02	1.38
**F-L1-05**	1	1.65	0.0036	22.02	9.35	1.28
**F-L1-06**	1	1.10	0.0032	23.44	9.47	1.30
**Average**	1	1.42 (0.284)	0.0031 (0.0006)	22.49 (0.820)	9.61 (0.357)	1.32 (0.049)
**F-L1-07**	2.5	8.51	0.0034	27.94	11.30	1.55
**F-L1-08**	2.5	6.55	0.0046	27.67	11.19	1.54
**F-L1-09**	2.5	8.54	0.0036	29.94	13.62	1.87
**Average**	2.5	7.87 (1.140)	0.0039 (0.0006)	28.52 (1.240)	12.04 (1.372)	1.65 (0.189)
**F-L1-10**	5	13.80	0.0017	27.23	12.40	1.70
**F-L1-11**	5	12.79	0.0031	28.15	12.81	1.76
**F-L1-12**	5	13.40	0.0036	23.59	10.73	1.47
**Average**	5	13.33 (0.508)	0.0028 (0.0009)	26.32 (2.411)	11.98 (1.101)	1.65 (0.151)
**F-L1-13**	6.5	17.80	0.0036	29.02	10.02	1.38
**F-L1-14**	6.5	13.35	0.0037	30.01	11.38	1.56
**F-L1-15**	6.5	20.01	0.0033	28.60	10.93	1.50
**Average**	6.5	17.05 (3.392)	0.0035 (0.0002)	29.21 (0.724)	10.78 (0.693)	1.48 (0.095)
**F-L2-01**	0.1	0.10	0.0016	31.30	12.08	1.33
**F-L2-02**	0.1	0.05	0.0019	30.81	11.80	1.30
**F-L2-03**	0.1	0.42	0.0037	30.48	11.77	1.30
**Average**	0.1	0.19 (0.201)	0.0024 (0.001)	30.86 (0.413)	11.88 (0.171)	1.31 (0.019)
**F-L2-04**	1	2.52	0.0023	31.57	12.19	1.35
**F-L2-05**	1	3.58	0.0029	35.53	13.71	1.51
**F-L2-06**	1	2.54	0.0031	28.70	11.07	1.22
**Average**	1	2.88 (0.606)	0.0028 (0.0004)	31.93 (3.429)	12.32 (1.325)	1.36 (0.146)
**F-L2-07**	2.5	5.01	0.0016	29.89	11.54	1.28
**F-L2-08**	2.5	5.65	0.0030	28.58	11.04	1.22
**F-L2-09**	2.5	4.58	0.0033	30.92	11.94	1.32
**Average**	2.5	5.08 (0.538)	0.0026 (0.0009)	29.80 (1.173)	11.51 (0.451)	1.27 (0.050)
**F-L2-10**	5	10.22	0.0027	29.83	11.52	1.27
**F-L2-11**	5	14.93	0.0023	31.14	12.02	1.33
**F-L2-12**	5	17.89	0.0016	29.84	11.52	1.27
**Average**	5	14.35 (3.868)	0.0022 (0.0006)	30.27 (0.753)	11.69 (0.289)	1.29 (0.032)
**F-L2-13**	6.5	18.29	0.0019	36.02	13.90	1.54
**F-L2-14**	6.5	21.39	0.0013	49.09	18.95	2.09
**F-L2-15**	6.5	24.23	0.0020	34.10	13.15	1.45
**Average**	6.5	21.30 (2.971)	0.0017 (0.0004)	39.74 (8.157)	15.33 (3.154)	1.69 (0.349)

[Note] Values in parentheses are standard deviations.

**Table 6:** Experimental results of GFRP-confined PC under dynamic tensile loading.

Specimen No.	Velocity (m/s)	Strain Rate (s^−1^)	Maximum Strain	Maximum Load (kN)	Dynamic Splitting Tensile Strength (MPa)	DIF
**G-L1-01**	0.1	0.11	0.0030	24.03	9.88	1.28
**G-L1-02**	0.1	0.16	0.0030	20.07	8.24	1.07
**G-L1-03**	0.1	0.31	0.0038	24.47	10.05	1.30
**Average**	0.1	0.19 (0.104)	0.0033 (0.0005)	22.86 (2.423)	9.39 (1.000)	1.22 (0.129)
**G-L1-04**	1	1.40	0.0014	22.56	9.27	1.20
**G-L1-05**	1	4.44	0.0033	25.87	10.63	1.38
**G-L1-06**	1	2.75	0.0031	24.68	10.14	1.31
**Average**	1	2.86 (1.523)	0.0026 (0.001)	24.37 (1.677)	10.01 (0.689)	1.30 (0.089)
**G-L1-07**	2.5	12.55	0.0034	26.69	10.97	1.42
**G-L1-08**	2.5	8.24	0.0025	26.80	11.01	1.43
**G-L1-09**	2.5	8.39	0.0018	29.73	12.22	1.58
**Average**	2.5	9.73 (2.446)	0.0026 (0.0008)	27.74 (1.724)	11.40 (0.710)	1.48 (0.092)
**G-L1-10**	5	10.91	0.0042	27.78	11.41	1.48
**G-L1-11**	5	15.35	0.0038	39.00	16.02	2.08
**G-L1-12**	5	15.61	0.0041	34.77	14.29	1.85
**Average**	5	13.96 (2.642)	0.0040 (0.0002)	33.85 (5.666)	13.91 (2.329)	1.80 (0.302)
**G-L1-13**	6.5	17.88	0.0038	33.05	13.58	1.76
**G-L1-14**	6.5	20.22	0.0041	32.07	13.18	1.71
**G-L1-15**	6.5	16.61	0.0033	29.00	11.92	1.54
**Average**	6.5	18.24 (1.831)	0.0037 (0.0004)	31.37 (2.113)	12.89 (0.866)	1.67 (0.112)
**G-L2-01**	0.1	0.32	0.0038	31.90	13.54	1.27
**G-L2-02**	0.1	0.61	0.0017	27.40	11.62	1.09
**G-L2-03**	0.1	0.26	0.0022	33.09	14.05	1.32
**Average**	0.1	0.40 (0.187)	0.0026 (0.001)	30.80 (3.001)	13.07 (1.281)	1.23 (0.121)
**G-L2-04**	1	2.72	0.0023	29.19	12.40	1.17
**G-L2-05**	1	0.62	0.0040	35.16	14.93	1.41
**G-L2-06**	1	1.70	0.0036	33.80	14.35	1.35
**Average**	1	1.68 (1.050)	0.0033 (0.0009)	32.72 (3.129)	13.89 (1.325)	1.31 (0.125)
**G-L2-07**	2.5	8.14	0.0034	38.30	16.26	1.53
**G-L2-08**	2.5	6.30	0.0033	33.42	14.19	1.34
**G-L2-09**	2.5	10.87	0.0040	38.46	16.33	1.54
**Average**	2.5	8.44 (2.299)	0.0036 (0.0004)	36.73 (2.865)	15.59 (1.216)	1.47 (0.114)
**G-L2-10**	5	23.22	0.0040	45.73	19.42	1.83
**G-L2-11**	5	21.06	0.0040	42.64	18.10	1.70
**G-L2-12**	5	23.24	0.0033	30.98	13.15	1.24
**Average**	5	22.51 (1.253)	0.0038 (0.0004)	39.78 (7.779)	16.89 (3.305)	1.59 (0.311)
**G-L2-13**	6.5	24.59	0.0032	39.49	16.77	1.58
**G-L2-14**	6.5	22.30	0.0032	37.70	16.01	1.51
**G-L2-15**	6.5	24.96	0.0039	33.04	14.03	1.32
**Average**	6.5	23.95 (1.441)	0.0034 (0.0004)	36.74 (3.330)	15.60 (1.415)	1.47 (0.133)

[Note] Values in parentheses are standard deviations.

## Data Availability

Part of the data underlying this article will be shared on reasonable request from the corresponding author.

## References

[1] Raza A., Shah S.A.R., Khan A.R., Aslam M.A., Khan T.A., Arshad K., Hussan S., Sultan A., Shahzadi G., Waseem M. (2020). Sustainable FRP-Confined Symmetric Concrete Structures: An Application Experimental and Numerical Validation Process for Reference Data. Appl. Sci..

[2] Pıhtılı H., Tosun N. (2002). Effect of load and speed on the wear behaviour of woven glass fabrics and aramid fibre-reinforced composites. Wear.

[3] Aniskevich K., Arnautov A., Jansons J. (2012). Mechanical properties of pultruded glass fiber-reinforced plastic after moistening. Compos. Struct..

[4] Lu H., Xu Z.-D., Iseley T., Matthews J.C. (2021). Novel Data-Driven Framework for Predicting Residual Strength of Corroded Pipelines. J. Pipeline Syst. Eng. Pract..

[5] Gao G., Li Y. (2016). Mechanical properties of woven glass fiber-reinforced polymer composites. Emerg. Mater. Res..

[6] Fanaradelli T.D., Rousakis T.C. (2020). Prediction of Ultimate Strain for Rectangular Reinforced Concrete Columns Confined with Fiber Reinforced Polymers under Cyclic Axial Compression. Polymers.

[7] Xu Z.-D., Yang Y., Miao A.-N. (2021). Dynamic Analysis and Parameter Optimization of Pipelines with Multidimensional Vibration Isolation and Mitigation Device. J. Pipeline Syst. Eng. Pract..

[8] Zaghloul M.M.Y., Zaghloul M.M.Y. (2017). Influence of flame retardant magnesium hydroxide on the mechanical properties of high density polyethylene composites. J. Reinf. Plast. Compos..

[9] Green M., Bisby L.A., Fam A.Z., Kodur V.K. (2006). FRP confined concrete columns: Behaviour under extreme conditions. Cem. Concr. Compos..

[10] Zaghloul M.M.Y.M. (2018). Mechanical properties of linear low-density polyethylene fire-retarded with melamine polyphosphate. J. Appl. Polym. Sci..

[11] Xu Z.-D. (2007). Earthquake Mitigation Study on Viscoelastic Dampers for Reinforced Concrete Structures. J. Vib. Control.

[12] Hollaway L.C., Chryssanthopoulos M., Moy S.S. (2004). Advanced Polymer Composites for Structural Applications in Construction: ACIC 2004.

[13] Zohrevand P., Mirmiran A. (2012). Cyclic Behavior of Hybrid Columns Made of Ultra High Performance Concrete and Fiber Reinforced Polymers. J. Compos. Constr..

[14] Xu Z.-D., Gai P.-P., Zhao H.-Y., Huang X.-H., Lu L.-Y. (2017). Experimental and theoretical study on a building structure controlled by multi-dimensional earthquake isolation and mitigation devices. Nonlinear Dyn..

[15] Wang W., Mo Z., Chouw N., Jayaraman K. (2021). Renovation Effect of Flax FRP-Reinforced Cracked Concrete Slabs under Impact Loadings. Materials.

[16] Tabatabaei Z.S., Volz J.S., Baird J., Gliha B.P., Keener D.I. (2013). Experimental and numerical analyses of long carbon fiber rein-forced concrete panels exposed to blast loading. Int. J. Impact Eng..

[17] Dai J., Xu Z.-D., Gai P.-P., Hu Z.-W. (2021). Optimal design of tuned mass damper inerter with a Maxwell element for mitigating the vortex-induced vibration in bridges. Mech. Syst. Signal Process..

[18] Liang J., Zou W., Li W., Liu D. (2021). Behaviour of CFRP strips confined partially encased concrete columns under axial com-pressive load. Compos. Struct..

[19] Wang J., Shen Q., Wang F., Wang W. (2018). Experimental and analytical studies on CFRP strengthened circular thin-walled CFST stub columns under eccentric compression. Thin-Walled Struct..

[20] Cao Q., Tao J., John Z., Wu Z. (2017). Axial Compressive Behavior of CFRP-Confined Expansive Concrete Columns. ACI Struct. J..

[21] Jin L., Li X., Fan L., Du X. (2020). Size Effect on Compressive Strength of GFRP-Confined Concrete Columns: Numerical Simulation. J. Compos. Constr..

[22] Pour A.F., Nguyen G.D., Vincent T., Ozbakkaloglu T. (2020). Investigation of the compressive behavior and failure modes of unconfined and FRP-confined concrete using digital image correlation. Compos. Struct..

[23] Djafar-Henni I., Kassoul A. (2018). Stress–strain model of confined concrete with Aramid FRP wraps. Constr. Build. Mater..

[24] Sun B., Liu X., Xu Z.-D. (2022). A Multiscale Bridging Material Parameter and Damage Inversion Algorithm from Macroscale to Mesoscale Based on Ant Colony Optimization. J. Eng. Mech..

[25] Yan L., Chouw N. (2013). Experimental study of flax FRP tube encased coir fibre reinforced concrete composite column. Constr. Build. Mater..

[26] Huang L., Liang J., Gao C., Yan L. (2021). Flax FRP tube and steel spiral dual-confined recycled aggregate concrete: Experimental and analytical studies. Constr. Build. Mater..

[27] Zaghloul M.Y.M., Zaghloul M.M.Y., Zaghloul M.M.Y. (2021). Developments in polyester composite materials–An in-depth review on natural fibres and nano fillers. Compos. Struct..

[28] Jirawattanasomkul T., Ueda T., Likitlersuang S., Zhang D., Hanwiboonwat N., Wuttiwannasak N., Horsangchai K. (2019). Effect of natural fibre reinforced polymers on confined compressive strength of concrete. Constr. Build. Mater..

[29] Gao C., Fu Q., Huang L., Yan L., Gu G. (2022). Jute fiber-reinforced polymer tube-confined sisal fiber-reinforced recycled aggregate concrete waste. Polymers.

[30] Fuseini M., Zaghloul M.M.Y. (2022). Qualitative and statistical approaches of the electrophoretic deposition kinetics of polyaniline copper coating. Prog. Org. Coat..

[31] Zaghloul M.M.Y., Zaghloul M.Y.M., Zaghloul M.M.Y. (2017). Experimental and modeling analysis of mechanical-electrical behaviors of polypropylene composites filled with graphite and MWCNT fillers. Polym. Test..

[32] Uddin N., Purdue J.D., Vaidya U. (2008). Feasibility of Thermoplastic Composite Jackets for Bridge Impact Protection. J. Aerosp. Eng..

[33] Xu Z.-D., Sun C.-L. (2021). Single-double chains micromechanical model and experimental verification of MR fluids with MWCNTs/GO composites coated ferromagnetic particles. J. Intell. Mater. Syst. Struct..

[34] Xiong B., Demartino C., Xiao Y. (2019). High-strain rate compressive behavior of CFRP confined concrete: Large diameter SHPB tests. Constr. Build. Mater..

[35] Chen Z., Huang L., Huang P., Xie J. (2022). Axial-Impact Resistance of CFRP-Confined Ultrahigh-Performance Concrete. J. Compos. Constr..

[36] Pham T.M., Hao H. (2017). Axial Impact Resistance of FRP-Confined Concrete. J. Compos. Constr..

[37] Huang L., Sun X., Yan L., Kasal B. (2017). Impact behavior of concrete columns confined by both GFRP tube and steel spiral reinforcement. Constr. Build. Mater..

[38] Yan Z.-W., Bai Y.-L., Ozbakkaloglu T., Gao W.-Y., Zeng J.-J. (2021). Axial impact behavior of Large-Rupture-Strain (LRS) fiber reinforced polymer (FRP)-confined concrete cylinders. Compos. Struct..

[39] Kheyroddin A., Arshadi H., Ahadi M., Taban G., Kioumarsi M. (2021). The impact resistance of Fiber-Reinforced concrete with polypropylene fibers and GFRP wrapping. Mater. Today Proc..

[40] Yang H., Song H., Zhang S. (2015). Experimental investigation of the behavior of aramid fiber reinforced polymer confined concrete subjected to high strain-rate compression. Constr. Build. Mater..

[41] Ge T., Xu Z.-D., Yuan F.-G. (2022). Predictive Model of Dynamic Mechanical Properties of VE Damper Based on Acrylic Rubber–Graphene Oxide Composites Considering Aging Damage. J. Aerosp. Eng..

[42] Song H., Yang H., Zhang S. (2017). Study on Dynamic Behavior of AFRP-Wrapped Circular Concrete Specimens under Repeated Impacts. Polym. Polym. Compos..

[43] Sawaki Y., Watanabe J., Nakanishi E., Isogimi K. (2006). Dynamic tensile behavior of aramid FRP using split hopkinson bar method. Fracture of Nano and Engineering Materials and Structures.

[44] Huang L., Gao C., Yan L., Yu T., Kasal B. (2018). Experimental and numerical studies of CFRP tube and steel spiral dual-confined concrete composite columns under axial impact loading. Compos. Part B Eng..

[45] Moustafa A., ElGawady M.A. (2016). Strain Rate Effect on Properties of Rubberized Concrete Confined with Glass Fiber–Reinforced Polymers. J. Compos. Constr..

[46] Huang L., Su L., Xie J., Lu Z., Li P., Hu R., Yang S. (2022). Dynamic splitting behaviour of ultra-high-performance concrete confined with carbon-fibre-reinforced polymer. Compos. Struct..

[47] Xie H., Yang L., Zhang Q., Huang C., Chen M., Zhao K. (2022). Research on energy dissipation and damage evolution of dynamic splitting failure of basalt fiber reinforced concrete. Constr. Build. Mater..

[48] Chen M., Zhong H., Wang H., Zhang M. (2020). Behaviour of recycled tyre polymer fibre reinforced concrete under dynamic splitting tension. Cem. Concr. Compos..

[49] Hassan M., Wille K. (2022). Direct tensile behavior of steel fiber reinforced ultra-high performance concrete at high strain rates using modified split Hopkinson tension bar. Compos. Part B Eng..

[50] Fu Q., Zhao X., Zhang Z., Peng G., Zeng X., Niu D. (2021). Dynamic splitting tensile behaviour and statistical scaling law of hybrid basalt–polypropylene fibre-reinforced concrete. Arch. Civ. Mech. Eng..

[51] Wang W., Chouw N. (2017). Behaviour of CFRC beams strengthened by FFRP laminates under static and impact loadings. Constr. Build. Mater..

[52] Yan L., Chouw N. (2013). Crashworthiness characteristics of flax fibre reinforced epoxy tubes for energy absorption application. Mater. Des..

[53] Wang W., Zhang X., Mo Z., Chouw N., Li Z., Xu Z.-D. (2020). A comparative study of impact behaviour between natural flax and glass FRP confined concrete composites. Constr. Build. Mater..

[54] Wang W., Mo Z., Chouw N., Jayaraman K. (2022). Strain rate effect on tensile strength of glass fiber-reinforced polymers. Int. J. Mod. Phys. B.

[55] Wang W., Zhang X., Chouw N., Li Z., Shi Y. (2018). Strain rate effect on the dynamic tensile behaviour of flax fibre reinforced polymer. Compos. Struct..

[56] Feng L., Chen X., Zhang J., Chen C. (2022). Experimental and mesoscopic investigation of self-compacting rubberized concrete under dynamic splitting tension. J. Build. Eng..

[57] Hao Y., Hao H. (2016). Finite element modelling of mesoscale concrete material in dynamic splitting test. Adv. Struct. Eng..

[58] Chen L., Yue C., Zhou Y., Zhang J., Jiang X., Fang Q. (2021). Experimental and mesoscopic study of dynamic tensile properties of concrete using direct-tension technique. Int. J. Impact Eng..

[59] (2010). Standard Test Method for Compressive Strength of Cylindrical Concrete Specimens.

[60] (2011). Standard Test Method for Splitting Tensile Strength of Cylindrical Concrete Specimens.

[61] Comité Euro-International du Béton (1993). CEB-FIP Model Code 1990.

[62] Malvar L.J., Ross C.A. (1998). Review of strain rate effects for concrete in tension. ACI Mater. J..

